# Impact of Recycled Fine Aggregate on Physical and Mechanical Properties of Green Mortar

**DOI:** 10.3390/ma18030696

**Published:** 2025-02-05

**Authors:** Xiaoqi Wan, Zhiyou Jia, Nannan Li, Hua Luo

**Affiliations:** 1 GCM—Joint Laboratory of Green Construction Materials, Department of Civil Engineering, Nanchang Institute of Technology, Nanchang 330013, China; 2 C-TAC—Centre for Territory, Environment and Construction, Department of Civil Engineering, University of Minho, 4800-058 Guimarães, Portugal; jiangdalinannan@gmail.com

**Keywords:** recycled fine aggregate, physical properties, mortar

## Abstract

Through research that combined green environmental protection with the resource usage of solid waste, we explored more possibilities for mortar using recycled fine aggregate (RFA) as a material. In this work, natural fine aggregate (NFA) with different proportions of RFA in mortar was produced, while maintaining the same particle size distribution. Four types of mortar were produced, with replacement ratios of 25%, 50%, 75%, and 100%, as well as a reference mortar type without RFA. A comprehensive evaluation of the mortar with different proportions was conducted, including its workability, density, capillary water absorption, compressive strength, and flexural strength. The results indicated that the compressive strength and flexural strength of mortar containing 50% RFA improve within 14 days. In addition, with increased RFA usage, the mortar’s mechanical properties decreased. The data obtained from this study will help in the application of RFA in green mortar.

## 1. Introduction

The demand for construction sand is growing at an astonishing rate because of the continued development of the construction industry, with estimates suggesting that by 2030, the annual demand for sand will reach 2 to 4.9 billion tons [1,2,3,4]. Natural river sand has been primarily used to produce concrete, accounting for more than 95% of its applications [5]. Wang et al. [2] found that China’s total sand supply increased nearly fivefold, rising from approximately 1.4 billion tons per year in 1995 to around 7.0 billion tons per year in 2020. Furthermore, the primary source of sand shifted from natural sand to manufactured sand. Over the past 20 years, Singapore has imported a total of 517 million tons of sand to achieve a 20% expansion in its land area [5]. The enormous demand for construction sand has led to the depletion of sand resources [1,6]. Finding alternatives to sand has become urgent [7].

A large amount of construction and demolition waste (CDW) has been generated due to the development of urban economies and the construction industry. Statistics from the Global Cement and Concrete Association show that the total quantity of CDW in China, the United States, and the European Union (28 EU countries) was estimated to be around 600 million tons in 2018 and increased to 807 million tons in 2020. Many scholars have researched the different applications of recycled aggregate from CDW to reduce the damage caused by CDW [8,9].

Among the various applications of CDW, recycled fine aggregates (RFAs) produced from CDW are commonly researched and adopted. RFA can be crushed and separated from waste construction materials, such as concrete, mortar, stone, bricks, and tiles [8,9], and it can partly replace natural fine aggregate, which is the main raw material in concrete, mortar, and other construction materials [6,7]. In addition, it is reused as a raw material for new materials [8,9,10].

Extensive research has been conducted on recycled-fine-aggregate concrete [11,12], mainly focusing on the impact of replacing natural fine aggregates with different proportions of RFAs on the properties of concrete [13,14]. As the amount of RFA increases, some properties show a significant downward trend [8,15,16]. Bogas et al. [17] studied the performance of concrete with the incorporation of different concentrations of RFA (0%, 20%, 50%, and 100%). The results showed that the compressive strength decreased as the replacement rate increased, especially in high-strength recycled concrete. Shah et al. [18] replaced natural fine aggregates in concrete with RFAs and investigated the impact of different replacement rates, ranging from 0 to 100%, on the performance of concrete. The study also observed the compressive strength, tensile strength, resistance to chloride ion penetration, resistance to chemical exposure for different curing times, and quality of the aggregates. The results indicated that using 100% fresh NFA in recycled aggregate concrete decreased the compressive strength, tensile strength, elastic modulus, and workability by approximately 30%, 35%, 20%, and 79%, respectively, compared to control concrete. However, according to the analysis of the results, under the suggested conditions and mix design approaches, the performance of recycled aggregate concrete is reasonable when 50% of NFA is replaced with recycled aggregates. Erhan et al. [19] investigated the rheological and fresh properties of self-compacting concrete using recycled concrete aggregates (RCAs) as coarse and fine aggregates. In this work, first, different mixing proportions were designed by replacing cement with 20% fly ash; replacing natural fine aggregate with 0%, 25%, 50%, 75%, and 100% RFA; and replacing natural coarse aggregate with 0%, 50%, and 100% recycled coarse aggregate. The results showed that the self-compacting performance of concrete can be significantly improved. These results have also been confirmed in other studies [20,21,22].

In addition, researchers have improved the performance of recycled aggregate concrete by incorporating different materials and chemical additives and optimizing mix design methods [14,15,16]. Sindhurashmi et al. [23] studied the use of undesorbed sea sand as fine aggregate to prepare self-compacting concrete. The results demonstrated that self-compacting concrete containing 50% sea sand and 50% manufactured sand showed excellent performance in terms of compressive strength on the 90th day. Ahmed et al. [24] studied the effects of recycled gravel and sand, as well as the initial water saturation of recycled aggregates, on plastic shrinkage and cracking sensitivity. The results showed that the proportion of recycled aggregate has a strong impact on cracking sensitivity, such as the concrete containing 30% recycled sand showing higher plastic shrinkage. Marija et al. [25] reviewed and summarized the latest research on RFA, including the engineering properties, physical and chemical properties, and durability of concrete containing recycled fine aggregate. Ahmed et al. [26] studied RFA and plastic waste to produce fine aggregate and prepare recycled-fine-aggregate concrete. Different proportions of silica fume, recycled coarse aggregate, and plastic waste could replace cement, natural coarse aggregate, and sand, respectively. As per the results, when the amount of plastic waste and RCA increases, the compressive strength, ultrasonic pulse velocity, and density decrease.

In recent studies, RFA has been mainly added to recycled concrete as a substitute for natural aggregate, and RFA concrete has been investigated for its physical, mechanical, and durability properties. It has also been used as a partial substitute to prepare mortar, and the performance of the mortar prepared with different types of single waste materials has been studied. In addition, various treatments that optimize the performance of mortar have been discussed [27,28]. Paswan et al. [29] studied the influence of incorporating microencapsulated phase-change materials into mortar on the mechanical behavior and evolution of microstructural damage under long-term freeze–thaw conditions. The results revealed that mortar with a higher concentration of microencapsulated phase-change materials exhibits less internal damage and maintains better mechanical integrity. Aboutaleb et al. [30] studied the potential of refractory bricks to replace fine aggregates in self-compacted mortar. After a brick was broken, the natural sand was replaced in different proportions (10%, 30%, 50%, and 100%). It was found that the fine aggregate in self-compacting mortar can be replaced with refractory bricks (0/5 mm) without affecting the basic properties of the mortar. Nevertheless, mortars containing 100% refractory bricks as RFA perform better and are suitable for use in flowable concrete. Minjae et al. [31] evaluated the impact of glass fine aggregate on mortar. The properties of glass fine aggregate and the effects of color, content, and particle size on the performance of mortar were analyzed. It was found that a high aspect ratio and micro-cracks within the particles lead to a reduction in the strength of the mortar and an increase in alkali–silica reaction expansion. It was proposed that the content of glass fine aggregate should be controlled within 20% or that fine particles (<500 μm) in natural fine aggregate should be replaced as conditions for sustainable fine aggregate. Vanderschelden et al. [32] studied the advantages of lime mortar in enhancing material reuse, reducing carbon dioxide emissions, and improving hygrothermal performance. The authors discussed lime mortar having lower carbon dioxide emissions compared to cement mortar. Zhu et al. [33] found that the workability, mechanical properties, and frost resistance of mortar are affected by the modulus of the alkali activator, glass fiber, and polypropylene fiber. Jia et al. [34] studied coffee grounds, which were made into cement mortar additives to optimize mortar strength. The research indicated that the strength of mortar is the highest when the amount of coffee grounds is 1.1%. However, the greater the amount of coffee grounds added, the lower the workability of the cement mortar mixture. Rosa et al. [35] replaced 50% or 100% of NFA with nepheline syenite beneficiation waste and converted into fine aggregates. It was found that the replacement of 50% NFA with nepheline syenite beneficiation waste does not have any significant effect on mortar in terms of rheological properties.

In this work, RFA was used in mortar to replace natural sand. A control group (R0) and 25%, 50%, 75%, and 100% RFA were used instead of NFA for a total of five groups of mortar samples. The evaluation indexes included the workability of fresh mortar and the density, water absorption, capillary water absorption, compressive strength, and flexural strength of the hardened mortar. The experimental data were analyzed and discussed. The physical and mechanical properties of green mortar were affected by the amount of RFA added. The results of this experiment will provide data support for the application of RFA in mortar, reduce the dependence on NFA, and improve the recycling of CDW.

## 2. Materials and Methods

### 2.1. Materials

**Figure 1:**
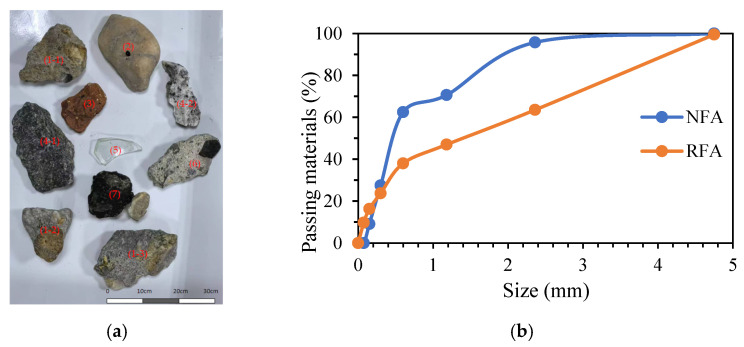
(**a**) Construction and demolition waste: (1-1, 1-2, 1-3) concrete waste, (2) cobblestone, (3) brick, (4-1, 4-2) natural stone, (5) glass, (6) mortar waste, and (7) asphalt concrete waste. (**b**) Size distribution of NFA and RFA.

In this work, ordinary Portland cement I 42.5 with a density of 3120 kg/m^3^ was used. The NFA was a type of commercial river sand from China. The density and water absorption of NFA with a size range of 0 to 4.75 mm were 2622.4 kg/m^3^ and 0.3%, respectively. The RFA was from CDW and included concrete waste, brick, natural stone, and glass (Figure 1). The original CDW was sourced from Nanchang Hengxin Building Materials Recycling, and its size ranged from 10 mm to 20 mm (Figure 1a). First, a jaw crusher was used to crush the CDW. After screening, the RFA obtained had a size distribution between 0 and 4.75 mm. The experimental methods in the GB/T 25176-2010 standard were used [36]. The density and water absorption of RFA were determined to be 2435.2 kg/m^3^ and 3.1%, respectively. Additionally, the size distribution of NFA and RFA was determined in accordance with the GB/T 14684-2022 standard [37], and the results are presented in Figure 1b. Furthermore, additional parameters are shown in Table 1. The water was tap water from the laboratory.

The particle size distribution of NFA was between 0 and 4.75 mm. The CDW was crushed and sieved, resulting in RFA with a size distribution of 0 to 4.75 mm. The difference between the two is shown in Figure 1b. It can be seen that the content of NFA was higher in the particle size range of 0.6–3.5 mm, while RFA was much less. This indicated that the particle size distribution of NFA is uniform, and the particle size distribution of RFA is uneven, with coarser particles.

### 2.2. Formulation Mortar Mix Ratio

The design of the reference mortar was prepared in accordance with the GB/T 17671-2021 standard [38]. The cement density and the water/cement ratio were set to 450 kg/m^3^ and 0.4, respectively. Five compositions were designed, 0%, 25%, 50%, 75%, and 100% RFA replacement of NFA (R0, R25, R50, R75, and R100, respectively). During the mortar-mixing process, the quantity of each material was first determined, with the water portion being divided into two parts: the water required for saturating the aggregates (absorbed water) and the water required for cement hydration and for guaranteeing the desired workability of mortars (effective water). Next, the fine aggregate and NFA were sequentially added to the mixer, along with the water, for saturating the aggregates, and mixed for 3 min to ensure uniform and complete wetting of the aggregates. Cement was then added, and mixing continued for 1 min to ensure even distribution of the cement with the aggregates. Finally, the water required for cement hydration and workability was slowly added, and the mixture was stirred for an additional 3 min. Afterward, the workability of the fresh mortar was tested. Additionally, the water/cement ratio of the five compositions was maintained at 0.4. Table 2 presents the materials used for all compositions.

### 2.3. Methods

#### 2.3.1. Consistency of Fresh Mortar

**Figure 2:**
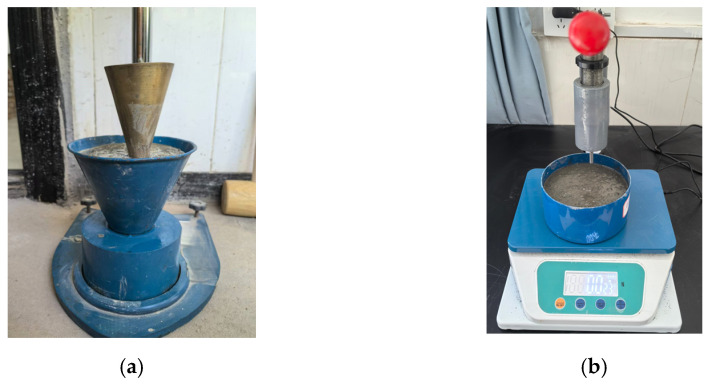
The workability of mortar: (**a**) consistency of fresh mortar and (**b**) setting time of fresh mortar.

All samples were prepared in a laboratory environment. The mortar consistency meter was used for measurement in accordance with the JGJ/T 70-2009 standard [39], as shown in Figure 2a. Initially, five groups of mortar samples (containing three samples each) with different mix proportions were mechanically mixed. Next, the samples were placed in containers, ensuring that the surface of the mortar was 10 mm below the edge of the container and the mortar remained compacted, and the containers with the samples were placed on the base of the consistency-testing apparatus. Furthermore, the mortar consistency-testing instrument was adjusted so that the tip of the test cone touched the surface of the mortar, and the locking screw was tightened, with the dial reading adjusted to zero. Finally, the locking screw was loosened and then tightened again after 10 s, ensuring that the bottom of the rack side rod touched the top of the sliding rod, and the dial reading was recorded. The mortar consistency test was repeated three times, and the mortar consistency value was calculated as the average of the three recorded readings.

#### 2.3.2. Setting Time of Fresh Mortar

The experimental methods in the JGJ/T 70-2009 standard [39] were used to ensure that the experimental steps were correct and the experimental results were reliable. At the same time, the entire experimental process was carried out at a temperature of 22 °C. The mortar setting time tester was used for measurement, as shown in Figure 2b.

First, five groups of mortar samples (containing three samples each) with different mix proportions were mechanically mixed. Next, the samples were placed in containers, ensuring that the surface of the mortar was 10 mm below the edge of the container, maintaining a smooth mortar surface, and the containers with the samples were placed on the base of the mortar setting time tester apparatus. Subsequently, the mortar setting time apparatus was adjusted using a penetration test needle with a cross-sectional area of 30 mm^2^, ensuring that the tip of the penetration test needle touched the surface of the mortar, and the reading was adjusted to zero. Finally, the mortar was vertically pressed to a depth of 25 mm within 10 s, and the reading was recorded. The mortar setting time test was repeated three times, and the penetration resistance value was calculated according to Formula (1) [39]. The mortar setting time value was calculated as the average of the three recorded readings.

(1) $$f_{p}=\frac{N_{p}}{A_{p}}$$

#### 2.3.3. Microstructure

Scanning electron microscopy (SEM) tests were used to examine the microstructure of mortar samples. Samples containing 0%, 25%, 50%, 75%, and 100% of RFA were tested. First, the mortar test blocks were cured in water for 28 days and dried to a constant weight. Second, the samples were crushed, cored, and fixed, with gold plating on the surface used as conductive particles. Finally, SEM tests were conducted, and the microstructure of the samples was observed [40].

#### 2.3.4. Density and Water Absorption of Mortar

The experimental methods in the GB/T 17671-2021 standard [38] were used, and the density and water absorption of the mortar test blocks were determined. First, the sample was dried at 105 °C for 24 h and then weighed as M_1_. Next, the sample was placed with the molding surface down into the water tank, held by two identical steel bars, ensuring that the sample was completely immersed in water and that the height from the top of the test block to the water surface was not less than 20 mm. The sample was immersed in water for 48 ± 0.5 h and then removed. A damp cloth, wrung out, was used to wipe away the surface moisture, and the sample was weighed as M_2_. Finally, the density and water absorption of the mortar were calculated according to Formulas (2) and (3).

(2) $$Dry\;density=\frac{M_{1}}{V}$$

(3) $$Water\;absorption=\frac{M_{2}-M_{1}}{M_{1}}\times 100\%$$

#### 2.3.5. Capillary Water Absorption of Mortar

The mortar samples were broken after flexural strength testing, the fracture surfaces were polished smooth, and mortar samples with dimensions of 80 mm × 40 mm × 40 mm were prepared. To begin with, the mortar samples were dried in an oven at a temperature of 105 ± 5 °C until a constant mass was obtained. The sides of the mortar samples were coated with epoxy resin at a height of 15 mm to ensure that water molecules did not penetrate.

**Figure 3:**
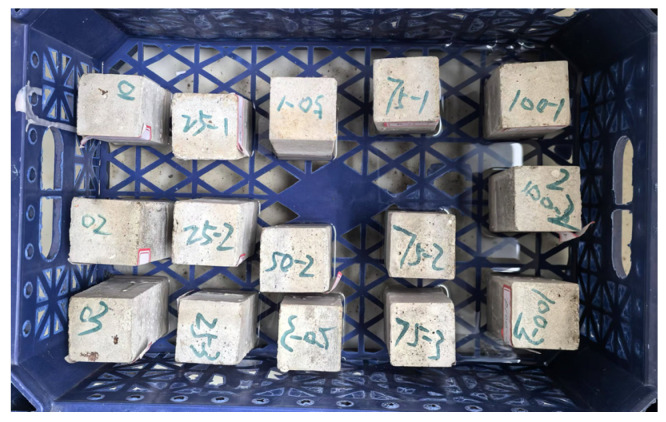
Capillary water absorption of mortar.

Next, the mortar samples were immersed in water to a depth of 10 mm, as shown in Figure 3, with the bottoms of the samples separated by grids and immersed in water, ensuring full contact with the water. Finally, a constant water level was maintained throughout the entire testing process. Mass M_1_ was measured after 10 min of immersion, mass M_2_ was measured after 90 min of immersion, and mass M_3_ was measured after 24 h of immersion. When the weight of the sample was stable, the test was stopped [41].

(4) $$Capillary\;water\;absorption\;coefficient=0.1\times \left(M_{2}-M_{1}\right)\;kg/\left(m^{2}\cdot min^{0.5}\right)$$

#### 2.3.6. Compressive Strength of Mortar

**Figure 4:**
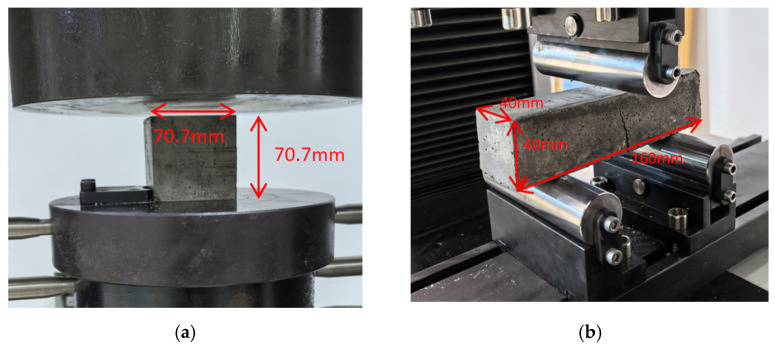
Mechanical properties of mortar: (**a**) mortar cube in compression test and (**b**) mortar sample during flexural test.

The experimental method in the JGJ/T 70-2009 standard [39] was used, and the compressive strength of mortar was tested after curing for 7, 14, and 28 days. The size of the mortar test block was 70.7 mm^3^. Figure 4a shows the compressive strength test of mortar samples. The mortar sample was placed on the lower platen of the testing machine, with the molded surface of the sample placed on the side, and the center of the sample was aligned with the center of the lower platen. A 0.25~1.5 kN/s pressure velocity was used, and the load was applied continuously and uniformly until failure. The maximum load of the mortar test block before failure divided by its cross-sectional area is equal to the compressive strength of the mortar.

#### 2.3.7. Flexural Strength of Mortar

The experimental method in the JC/T 724-2005 standard [42] was used, and the flexural strength was tested. The size of the mortar sample was 40 mm × 40 mm × 160 mm. All mortar test blocks were cured in water for 7, 14, and 28 days. The WDW-100E tensile testing machine (Hengda HSBC Testing Instrument Co., Ltd., Jinan, China) was used for the flexural strength test. The mortar sample was placed in the fixture of the testing instrument. The state of the sample in the fixture is shown in Figure 4b. The three-point flexural strength test was used; the load was uniformly applied at a slow loading rate of 10 N/s until the sample was damaged. The average value of three samples was used to calculate the flexural strength. The broken specimens were used in the capillary water absorption test.

## 3. Results and Discussion

### 3.1. Fresh Mortar Consistency

**Figure 5:**
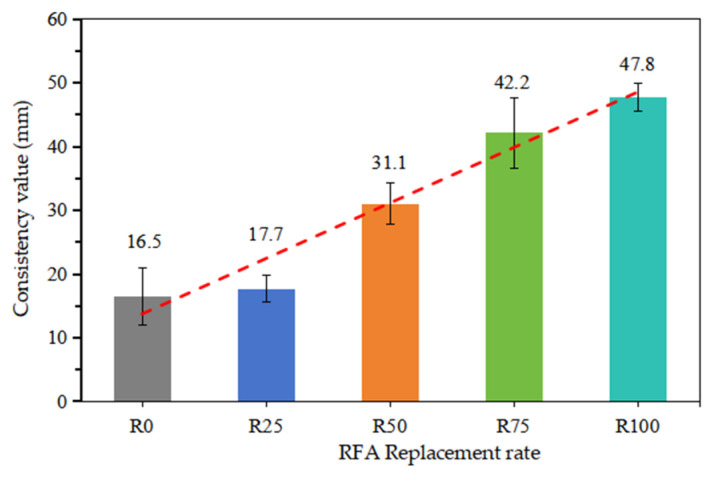
Consistency value of all compositions.

Figure 5 illustrates the results of the consistency value of fresh mortar. With RFA incorporation, the consistency value increased by 7.3%, 88.5%, 155.8%, and 189.7% for mortars R25, R50, R75, and R100, respectively, indicating that mortar becomes more fluid. This can be explained by the fact that RFA has more open pores than NFA, so part of the saturation water contributes to the workability of fresh mortar. As discussed by Majhi et al. [43], the greater water absorption capacity of RFA necessitates more absorbed water for saturating the aggregates, leading to a higher initial free-water content in the fresh mixture, since there are numerous open pores in RFA. This higher initial free-water content contributes to an increase in the workability of the fresh mixture, as noted in the study by Behera et al. [44]. Their study demonstrated that this type of higher initial free-water content does not adversely affect mixture strength.

### 3.2. Setting Time of Fresh Mortar

**Figure 6:**
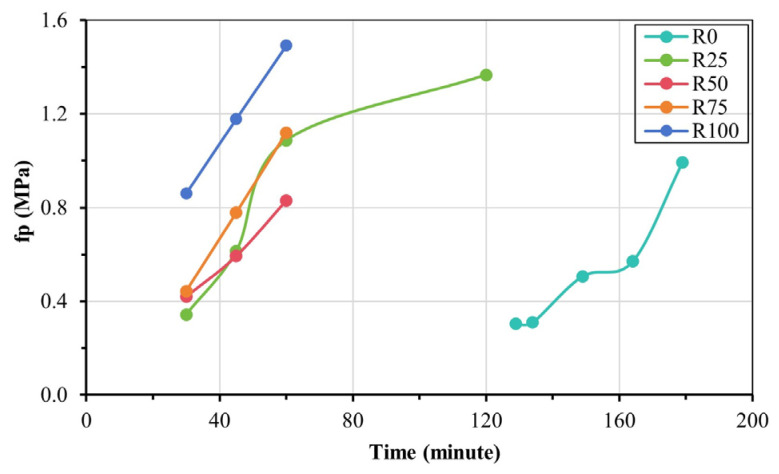
Setting time of fresh mortar.

The relationship between the penetration resistance value and time is shown in Figure 6. According to the JGJ/T 70-2009 standard [39], the setting time of mortar is the time corresponding to the penetration resistance value reaching 0.5 MPa, that is, the time when the mortar mixture solidifies and loses plasticity after the addition of water.

The setting time of fresh mortar mixed with different proportions of RFA is shown in Figure 6. The setting time of R0 was more than 2 h. With RFA incorporation, the penetration resistance values increased by 2.8%, 5.6%, 25%, and 138.9% at 30 min, respectively, for mortars R25, R50, R75, and R100, indicating an early increase in mortar’s strength and a faster setting time.

The setting time of fresh mortar is shorter. When the proportion of RFA is increased in the mortar mixture, the transition time of mortar is significantly shortened from the flow state to the hardening state. This is because the setting time of fresh mortar is affected by various factors, including its particle shape, porosity, density, and water/cement ratio. The proportion of RFA also has an important effect on the optimization of the setting time of the mortar mixture [45,46].

### 3.3. Microstructure Analysis of Mortar Samples

**Figure 7:**
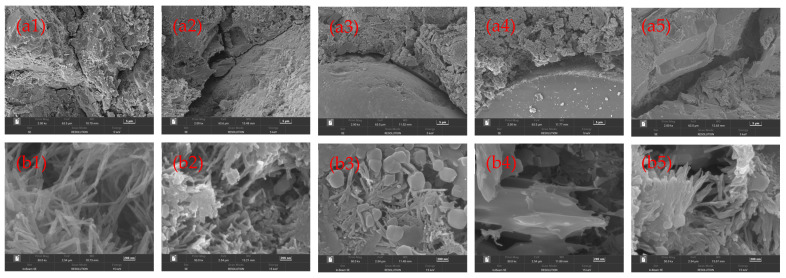
Microstructure of mortar samples using SEM: (**a1**–**a5**) R0, R25, R50, R75, and R100 2000× magnification scope; (**b1**–**b5**) R0, R25, R50, R75, and R100 50,000× magnification scope.

The SEM test results of the mortar mixtures with RFA are shown in Figure 7, displaying the SEM micrographs of the control mix (Figure 7(a1,b1)), the 25% replacement level (Figure 7(a2,b2)), the 50% replacement level (Figure 7(a3,b3)), the 75% replacement level (Figure 7(a4,b4)), and the 100% replacement level (Figure 7(a5,b5)) [47].

Calcium silica hydrate (C-S-H) gel and calcium hydroxide (CH) are the main hydration products in mortar [48]. In this study, clear C-S-H gel and CH were distributed all around R0. NFA and cement particles were closely combined without forming an interfacial transition zone. The microstructures of the five groups of mortar test blocks (R0, R25, R50, R75, and R100) were compared. It was observed that interfacial transition zones were formed between new mortar and old mortar, between new mortar and fine aggregate, and between old mortar and fine aggregate [49,50].

When the proportion of RFA increased, the C-S-H gel and CH changed. In the mortar mixtures (R25, R50, R75, and R100), the C-S-H gel and CH decreased and were unevenly distributed compared to mortar R0, and the number of pores in the mortar remarkably increased. An interfacial transition zone developed within the mortar mixture, which reduced the bond strength between RFA and the adhering mortar. However, the inclusion of RFA did not negatively affect the strength, as the strength of the mortar mixes was within limits and the mortars possessed comparable physical and mechanical properties [51,52].

### 3.4. Density and Water Absorption of Mortar

**Figure 8:**
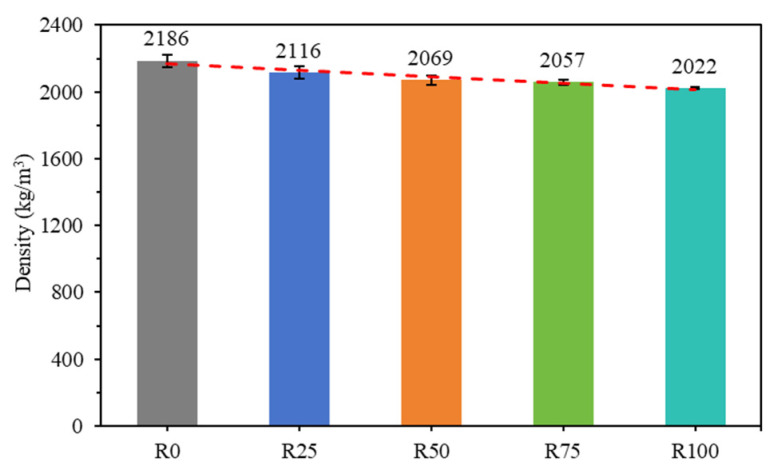
Density of all compositions.

The experimental methods according to the JGJ/T 70-2009 standard were used, and the density and water absorption of the mortar samples were determined [39]. The differences in dry density among the five groups of mortar mixtures are shown in Figure 8. The dry densities decreased by 3.2%, 5.3%, 5.9%, and 7.5%, respectively, for mortars R25, R50, R75, and R100, compared to mortar R0, indicating that mortar’s dry density is reduced as the incorporation of RFA increases. The apparent density and saturation density of NFA were both higher than those of RFA, as shown in Table 1, revealing that the density of mortar is affected by the composition of RFA and the proportion of addition [53].

**Figure 9:**
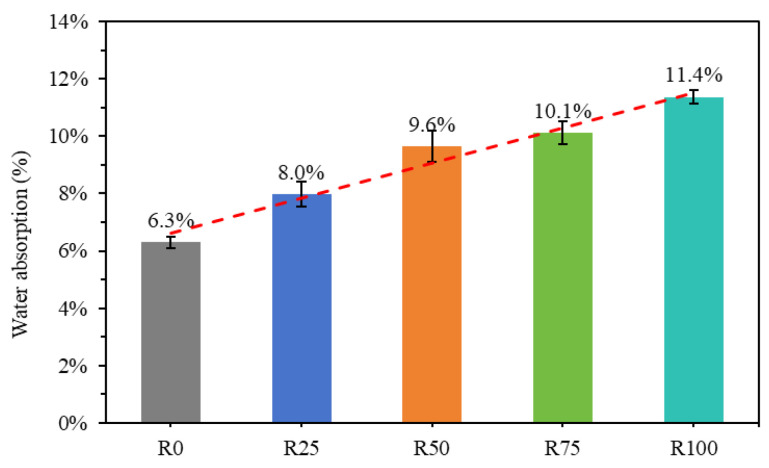
Water absorption of all compositions.

The water absorption test results of the mortar samples are shown in Figure 9. It was observed that the absorption rate of R0 was the lowest of all water absorption rates. Specifically, the water absorption rates increased by 26.7%, 53%, 60.4%, and 80.4%, respectively, for mortars R25, R50, R75, and R100, indicating that there are more pores in these mortar mixtures. Furthermore, the water absorption of RFA was significantly higher than that of NFA, as shown in Table 1. This explains why the higher the proportion of RFA added, the greater the water absorption of mortar. The results showed that the water absorption of mortar mixtures is influenced by the composition, porosity, and addition ratio of RFA, as noted in a study by Jia et al. [9]. As discussed by Jiang et al. [54], the addition of RFA to mortar may lead to the creation of porous structures.

### 3.5. Capillary Water Absorption of Mortar

**Figure 10:**
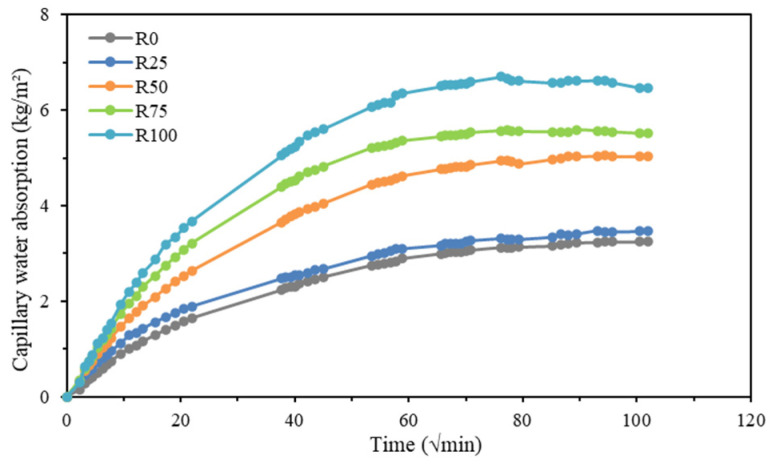
Capillary water absorption of all compositions.

The capillary water absorption of the five groups of mortar test blocks was tested, and the measurement results of water absorption were recorded at different time intervals over a period of 180 h according to the EN 1015-18:2002 standard [41]. As shown in Figure 10, the capillary water absorption curves differed significantly among the five groups of mortar mixtures.

**Figure 11:**
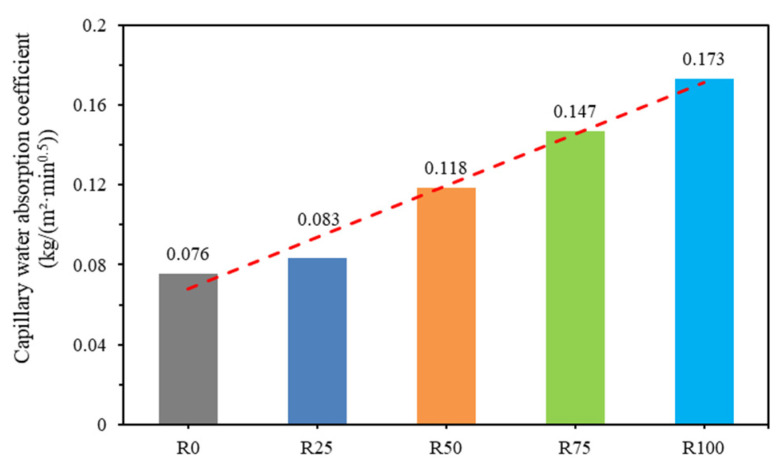
Capillary water absorption coefficients of mortar samples.

The capillary water absorption coefficients of mortar samples were calculated according to Formula (4), and the results are shown in Figure 11. Specifically, capillary water absorption increased by 26.7%, 53%, 60.4%, and 80.4%, respectively, for mortars R25, R50, R75, and R100, indicating that mortar workability improved significantly [9].

The results showed that the mortar mixtures (R25, R50, R75, and R100) had more voids, cracks, or surface capillary channels. Additionally, the R100 mortar mixture showed the highest capillary water absorption coefficient. According to the principle of capillary water absorption, the capillary water absorption coefficient increases because the existence of RFA aggravates the occurrence of the capillary phenomenon. As discussed in the studies by Yue et al. [55] and Yuanyu et al. [56], the addition of different proportions of RFA to mortar has a significant effect on the mortar’s capillary water absorption performance.

### 3.6. Compressive Strength of Mortar

**Figure 12:**
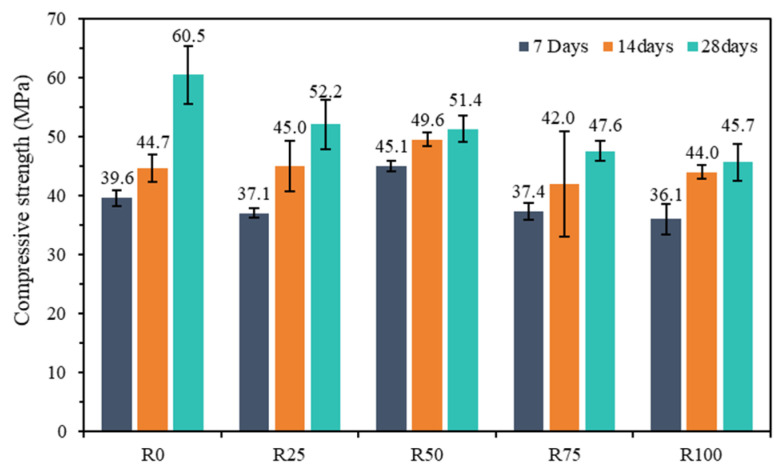
Compressive strength of all compositions.

Mortar cubes were cured in water for 7, 14, and 28 days, and their compressive strength was evaluated in accordance with the JGJ/T 70-2009 standard [39]; the results are shown in Figure 12.

The compressive strength decreased by 6.3%, 5.6%, and 8.8% at 7 days for mortars R25, R75, and R100, respectively, but that for mortar R50 increased by 13.9%. The compressive strength increased by 0.7% and 11% at 14 days for mortars R25 and R50, respectively, but decreased by 6.0% and 1.6% for mortars R75 and R100, respectively. The compressive strength decreased by 13.7%, 15%, 21.3%, and 24.5% at 28 days for mortars R25, R50, R75, and R100, respectively. The results indicated that the compressive strength of the mortar specimens is affected and that the compressive strength of the mortar mixture decreases at 28 days as the content of regenerated RFA increases.

The compressive strength of mortars R0, R25, R50, R75, and R100 increased by 52.8%, 40.7%, 14%, 27.3%, and 26.6%, respectively, at 28 days. This indicates that the compressive strength of mortar increases rapidly in the early stages as the incorporation of RFA increases but that the growth slows in the later period. As discussed in a study by Onuaguluchi et al. [57], the addition of different ratios of RFA to mortar has a significant influence on compressive strength.

### 3.7. Flexural Strength of Mortar

**Figure 13:**
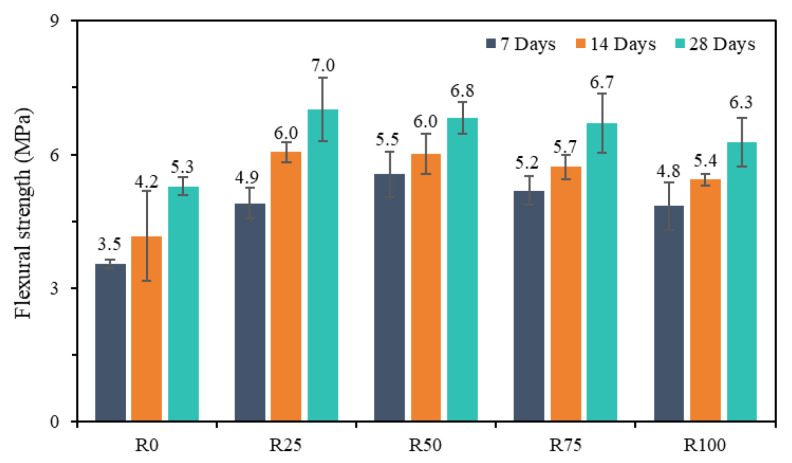
Flexural strength of all compositions.

The flexural strength test was performed following the JC/T 724-2005 standard [42], and the results are presented in Figure 13.

Compared to mortar R0, for mortars R25, R50, R75, and R100, flexural strength increased by 40%, 57.1%, 48.6%, and 37.1%, respectively, at day 7; by 42.9%, 42.9%, 35.7%, and 28.6%, respectively, at day 14; and by 32.1%, 28.3%, 26.4%, and 18.9%, respectively, at day 28. It is noteworthy that a significant improvement in flexural strength was observed when RFA was incorporated into the mortar mixture [9]. This result is attributed to the composition and particle properties of RFA, which exhibit higher reactivity and positively influence the cement hydration process. Therefore, it is necessary to optimize the mortar flexural strength at an early stage [58].

At 28 days, the flexural strength of mortars R0, R25, R50, R75, and R100 increased by 51.4%, 42.9%, 23.6%, 28.8%, and 31.3%, respectively. The results indicate that as the proportion of RFA increases, the rate of increase in flexural strength decreases. The results also reveal the flexural strength properties of mortar mixtures and highlight the potential effects of incorporating RFA into mortar on flexural strength [59].

## 4. Conclusions

In response to the global challenges of excessive construction demolition waste generation and overconsumption of NFA, this study explored the physical and mechanical properties of mortar when NFA is replaced with RFA. In this work, the application of RFA in mortar was studied with regard to the following aspects:

Physical properties of RFA: The density and water absorption of RFA were tested. The results suggest that the density of RFA is lower, and its water absorption is higher compared to NFA.

Consistency: The relationship between the consistency of fresh mortar and that of recycled fine aggregate was observed; the greater the amount of RFA, the greater the water requirement, ensuring the desired consistency.

Setting time: According to the observed relationship between the fresh mortar penetration resistance value and time, the higher the RFA proportion in the mortar, the faster the setting time.

Microstructure: The mortar incorporated with 25%, 50%, 75%, and 100% of RFA showed a decrease in and an uneven distribution of C-S-H gel and CH and a remarkable increase in the number of pores in the mortar mixtures. An interfacial transition zone developed within the mortar mixtures.

Physical properties of mortar: The density, water absorption, and capillary absorption of mortar are closely related to RFA. The density of mortar is low when the density of RFA is low. Mortar’s water absorption and capillary water absorption are high because RFA has higher water absorption. In particular, mortar with 100% RFA has the highest water absorption performance.

Mechanical properties of mortar: When the content of RFA is 25%, the mortar test blocks exhibit higher compressive and flexural strength values. However, the R100 mortar mixture displays the best compressive and flexural strength values. Considering that the aim of this work was to produce green mortars, mortars with 100% RFA have the best eco-efficiency.

## Figures and Tables

**Table 1:** Parameters of the fine aggregates used.

Aggregate	ρ_A_ (kg/m^3^)	ρ_s_ (kg/m^3^)	W_A_ (%)	FM
NFA	2614.7	2622.4	0.3	2.3
RFA	2300.5	2435.2	5.9	3.1

ρ_A_: apparent density; ρ_s_: saturation density; W_A_: water absorption; FM: fineness modulus.

**Table 2:** Mortar mix ratio of 1 m^3^.

Type	Cement (kg/m^3^)	Water (kg/m^3^)	NFA0-4.75 mm (kg/m^3^)	RFA0-4.75 (kg/m^3^)	Saturated Water (kg/m^3^)
R0	450	180	1350	0.00	3.92
R25	450	180	1012.5	296.94	20.34
R50	450	180	675	593.87	36.76
R75	450	180	337.5	890.81	53.18
R100	450	180	0	1187.75	69.60

## Data Availability

The original contributions presented in this study are included in the article. Further inquiries can be directed to the corresponding author.
